# Value of psychological counseling for trainees exposed to the death of a patient in emergency and resuscitation departments

**DOI:** 10.1192/j.eurpsy.2022.1729

**Published:** 2022-09-01

**Authors:** I. Betbout, B. Amemou, A. Ben Haouala, Y. Ben Arbia, I. Khadhrawi, F. Zaafrane, A. Mhalla, L. Gaha

**Affiliations:** 1 fattouma bourguiba hospital, Psychiatry, Psychiatry Research Laboratory Lr05es10 “vulnerability To Psychoses” Faculty Of Medicine Of Monastir, University Of Monastir, monastir, Tunisia; 2 High school of sciences and techniques of the health of Sousse, Psychiatry, sousse, Tunisia

**Keywords:** psychological reactions-trainee -death of a patient

## Abstract

**Introduction:**

Trainee emergency and resuscitation technicians are not prepared during their academic training to deal with their psychological reactions to the death of a patient, we wanted to describe their feelings and understand the aggravating factors and highlight the need for intervention.

**Objectives:**

Our study aims to describe the psychological reactions of traineesexposed to the death of a patient on the internship grounds and to demonstrate the usefulness of specific psychological counseling

**Methods:**

It is a prospective interventional study carried out with 2nd and 3rd-year students of the emergency and resuscitation section, our collection was done using a self-administered questionnaire with a validated PDI scale before the training, and a satisfaction questionnaire with the same scale after the training.

**Results:**

Our population isyoung, with an averageage of 20.05 years, and ispredominantlyfemale, with a sex ratio of 0.12. Eighty-seven percent of the population statedthatthey were not prepared to deal with their feelings about the death of a patient, and thiseventharmed the quality of care for 68% of the students. According to the scores of the PDI scale in pre-training 77.33% of the students are at risk of developing PTSD, this percentage decreases to 30.67% according to the same scale in post-training.

**Conclusions:**

it is important to take into consideration the suffering of traineesexposed to traumaticevents such as the death of patients and to prepare them psychologically to deal with these situations

**Disclosure:**

No significant relationships.

